# Stage of HIV presentation at initial clinic visit following a community-based HIV testing campaign in rural Kenya

**DOI:** 10.1186/s12889-015-1367-4

**Published:** 2015-01-21

**Authors:** John Haskew, Kenrick Turner, Gunnar Rø, Andrew Ho, Davies Kimanga, Shahnaaz Sharif

**Affiliations:** Uamuzi Bora, PO Box 2153-50100, Kakamega, Kenya; British Antarctic Survey Medical Unit, Plymouth, UK; University of Durham, Durham, UK; East and North Hertfordshire NHS Trust, London, UK; Elizabeth Glazer Paediatric AIDS Foundation, Nairobi, Kenya; Ministry of Health, Nairobi, Kenya

**Keywords:** Electronic medical record, Resource-constrained settings, Community-based HIV testing, HIV testing, Linkage to care

## Abstract

**Background:**

The Kenyan Ministry of Health and partners implemented a community-based integrated prevention campaign (IPC) in Western Kenya in 2008. The aim of this study was to determine whether the IPC, compared to Voluntary Counselling and Testing (VCT) services, was able to identify HIV positive individuals earlier in the clinical course of HIV infection following testing.

**Methods:**

A total of 1,752 adults aged over 15 years who tested HIV positive through VCT services or the IPC, and subsequently registered at initial clinic visit between September 2008 and September 2010, were considered in the analysis. Multivariable logistic regression models were developed to assess the association of CD4 count and WHO clinical stage of HIV infection at first clinic appointment with age group, gender, marital status and HIV testing source.

**Results:**

Male gender and marital status were independently associated with late HIV presentation (WHO clinical stage 3 or 4 or CD4 count ≤350 cells/μl) at initial clinic visit. Patients testing HIV positive during the IPC had significantly higher mean CD4 count at initial clinic visit compared to individuals who tested HIV positive via VCT services. Patients testing HIV positive during the IPC had more than two times higher odds of presenting early with CD4 count greater than 350 cells/μl (adjusted OR 2.15, 95% CI 1.28 – 3.61, p = 0.004) and presenting early with WHO clinical stage 1 or 2 of HIV infection (adjusted OR 2.39, 95% CI 1.24 – 4.60, p = 0.01) at initial clinic visit compared to individuals who tested HIV positive via VCT services.

**Conclusion:**

The community-based integrated prevention campaign identified HIV positive individuals earlier in the course of HIV infection, compared to Voluntary Counselling and Testing services. Community-based campaigns, such as the IPC, may be able to assist countries to achieve earlier testing and initiation of ART in the course of HIV infection. Improving referral mechanisms and strengthening linkages between HIV testing and treatment services remain a challenge and electronic medical record (EMR) systems may support monitoring of patients throughout the HIV care and treatment continuum.

## Background

The Joint United Nations Programme on HIV/AIDS (UNAIDS) estimates 35.3 million people worldwide are living with HIV, with an estimated 2.3 million new infections in 2012 [1]. The epidemic continues to disproportionately affect sub-Saharan Africa, which is home to 70% of all new HIV infections [1]. Knowledge of HIV status remains inadequate, despite significant scaling up of HIV testing services over the past decade [1]. In 2011, an estimated 36% of people in the region had never been tested for HIV and less than 50% of people living with HIV in sub-Saharan Africa knew their status [1,2].

Due to the lack of access to HIV testing services, an estimated 25% of people who initiate ART in low- and middle-income countries have CD4 counts <100 cells/μl, and late stage of HIV presentation at the time of ART initiation is an important prognostic factor for treatment success [3]. Patients with advanced HIV disease at the time of starting ART, defined by low CD4 count and World Health Organization (WHO) clinical stage 3 or 4 of infection, are less likely to respond to treatment and have a higher mortality rate compared with those who start treatment earlier [4-6]. In addition, those who survive suffer more morbidity and utilise more health care resources than would otherwise have been necessary, placing increased financial burden on health services [7]. Late presentation also poses a higher cumulative risk of HIV transmission to others, considering that earlier presentation and access to ART can reduce viral load and risk of onward transmission [6,8-10].

In an effort to increase access to HIV testing services, and to identify people living with HIV earlier in the course of infection, the Kenyan Ministry of Health and partners launched a community integrated prevention campaign (IPC) in Western Province, Kenya in September 2008 [11]. The IPC provided HIV testing and counseling services, water filters, insecticide-treated bed nets, condoms, and for HIV-infected individuals cotrimoxazole prophylaxis and referral to care. 47,311 people attended the campaign, achieving 87% coverage among the target population over the age of 15 years in seven days and, of all those tested, 1,956 (4.1%) were found to be HIV positive and referred to Ministry of Health care and support services [11].

There is a paucity of published data comparing linkage to HIV treatment services from different sources of HIV testing. The overall aim of this study was to determine whether the IPC, compared to Voluntary Counselling and Testing (VCT) services, was able to identify HIV positive individuals earlier in the clinical course of infection, defined by CD4 count and WHO clinical stage of HIV infection at first clinic visit.

## Methods

### Study population and design

Data for this study included all patients who attended the HIV clinic of Kakamega Provincial General Hospital, Western Kenya for their first clinic visit between January 2004 and September 2010. In 2012, HIV prevalence in Western Kenya is 4.7%, compared to a national average of 5.6% [12].

Data were obtained from the Uamuzi Bora Electronic Medical Record (EMR) system [13], an open-source web-based database that replicates information collected in the national paper-based HIV outpatient record, which includes socio-demographic information, patient source of testing, treatment history, and clinical and laboratory results. The EMR system was designed to enable the Ministry of Health to maintain accurate HIV medical records, track patients and monitor outcomes more effectively.

Clinical and demographic data from the initial clinic visit were extracted for use in the analysis, including age, gender, source of HIV testing, date of diagnosis, date of first clinic appointment, clinical data from first clinic appointment (WHO clinical stage of infection) and date started ART. Quality control checks were performed during data entry into the EMR database from the paper record, during which electronic records were compared variable by variable with the original paper record.

In September 2010, 6,657 HIV positive patients were registered in the EMR system, including 108 adults who tested HIV positive during the IPC and 3,651 patients who tested HIV positive via VCT services.

### Statistical analysis

All analyses were carried out using STATA version 12.1 (Stata Corp; College Station, TX). Primary outcomes considered for analysis were CD4 count and WHO clinical stage of HIV infection at initial clinic visit.

Early or late presentation of HIV (by CD4 count or WHO clinical stage of HIV infection) was defined according to thresholds for initiation of antiretroviral therapy (ART) as indicated in the 2011 fourth edition of the Guidelines for antiretroviral therapy in Kenya [14]. This guideline recommends ART to be started in all HIV-positive adults and adolescents with WHO clinical stage 1 or 2 and a CD4 count ≤ 350 cells/μl or with WHO clinical stage 3 or 4 regardless of CD4 count. CD4 count was coded as a numeric value at initial clinic visit and as a binary variable, defined as early presentation (CD4 > 350 cells/μl) or late presentation (CD4 ≤ 350 cells/μl) according to the 2011 Guidelines for antiretroviral therapy in Kenya [14]. WHO clinical stage of HIV infection was categorised as early presentation (stages 1 or 2) or late presentation (stages 3 or 4) according to 2011 Guidelines for antiretroviral therapy in Kenya [14]. Age group, gender, marital status and HIV testing source were considered independent variables in the analysis.

A total of 3,759 adults testing HIV positive through VCT services (n = 3,651) or the IPC (n = 108) were initially considered in the analysis. VCT patients prior to 1 September 2008 were excluded from analysis (n = 1,944), because the IPC was undertaken in September 2008 and improvements in provision of HIV testing and treatment services since the first patients were registered at the clinic in 2004 could bias results. All children under the age of 15 years were also excluded from VCT services (n = 63) because the IPC only included individuals over the age of fifteen, leaving a total sample size of 1,752 considered for analysis (1,644 from VCT, 108 from IPC). Patients with missing data for WHO clinical stage and CD4 count were compared to patients with complete data using Pearson χ2 tests to check for differences in distribution of socio-demographic variables and to assess for the likelihood of bias.

A multivariable logistic regression model was developed to identify independent correlates of late presentation. The model was built in a step-wise fashion, initially including HIV testing source and potential confounders showing a strong association with the outcome (p < 0.1) in univariable analysis. The variable most strongly related (lowest p-value) to the outcome in univariable analysis was the first to be included in the logistic regression model. A likelihood ratio test was then carried out on the model with and without this variable. The variable with the next smallest *P*-value from the univariable analyses was then added into the model. Variables were added until no further variables improved the fit of the model (p > 0.05). Any variables with p > 0.05 were tested to ensure they had no effect on the final multivariable model. Adjusted multivariable odds ratios, 95% confidence intervals and p-values are presented.

### Ethical issues

The study was approved by the institutional review board at Kakamega Provincial General Hospital and the Director of Public Health, Ministry of Health, Kenya.

## Results

### Missing data

Of 1,752 patient records initially included in the analysis, just over one half did not record CD4 count and one quarter of patient records did not record WHO clinical stage on the initial visit form (Table 1). Patients who had CD4 count and WHO clinical stage information were compared with those who had none using Pearson χ^2^ trend tests and significant difference (p < 0.05) was found between different patient sources of HIV testing source (Table 1). Patients with missing CD4 count and WHO clinical stage were therefore removed from subsequent analysis when comparing IPC to VCT services (Tables 2 and 3).

**Table 1:** **Socio-demographic characteristics of patients with and without initial WHO clinical stage and CD4 count recorded**

**Characteristic**	**N. (%) with initial WHO stage**	**N. (%) missing initial WHO stage**	**Pearson χ** ^**2**^	***P***	**N. (%) with initial CD4 count**	**N. (%) missing initial CD4 count**	**Pearson χ** ^**2**^	***P***
Total	1,314 (75.0%)	438 (25.0%)			861 (49.1%)	891 (50.9%)		
Patient testing source			4.263	0.04			14.14	0.000
IPC	90 (86.5%)	18 (16.7%)			72 (66.7%)	36 (33.3%)		
VCT	1,224 (74.5%)	420 (25.5%)			789 (48.0%)	855 (52.0%)		
Age group			1.416	0.23			0.087	0.77
50 years and under	1,106 (74.5%)	379 (25.5%)			732 (49.3%)	753 (50.7%)		
Over 50 years	208 (77.9%)	59 (22.1%)			129 (48.3%)	138 (51.7%)		
Gender			0.515	0.47			1.406	0.24
Female	833 (74.4%)	286 (25.6%)			538 (48.1%)	581 (51.9%)		
Male	481 (76.0%)	152 (24.0%)			323 (51.0%)	310 (49.0%)		
Marital status^*^			8.468	0.01			1.06	0.59
Married	691 (76.5%)	212 (23.5%)			444 (49.2%)	459 (50.8%)		
Never married	125 (66.5%)	63 (33.5%)			85 (45.2%)	103 (54.8%)		
Divorced/widowed	368 (75.6%)	119 (24.4%)			240 (49.3%)	247 (50.7%)		

^*^174 missing values.

**Table 2:** **Socio-demographic, clinical characteristics and univariable/multivariable associations with late HIV disease presentation (WHO clinical stage 3 or 4) at initial clinic visit**

**Characteristics**	Early presentation (WHO stage 1/2) N. (%)*	Late presentation (WHO stage 3/4) N. (%)*	**Unadj OR (95% CI)**	***P***	**Adj OR** ^**^**^ **(95% CI)**	***P***
All participants	955 (72.7%)	359 (27.3%)				
HIV testing source						
IPC	75 (83.3%)	15 (16.7%)	1			
VCT	880 (71.9%)	344 (28.1%)	1.95 (1.11 – 3.45)	0.02	2.39 (1.24 – 4.60)	0.01
Age						
50 years and under	809 (73.1%)	297 (26.9%)	1		1	
Over 50 years	146 (70.2%)	62 (29.8%)	0.46 (0.28 – 0.74)	0.002	1.10 (0.77 – 1.57)	0.61
Gender						
Female	623 (74.5%)	210 (25.2%)	1		1	
Male	332 (69.0%)	149 (31.0%)	1.33 (1.04 – 1.71)	0.02	1.38 (1.04 – 1.83)	0.02
Marital status^†^						
Married	524 (75.8%)	167 (24.2%)	1		1	
Never married	95 (76.0%)	30 (24.0%)	0.99 (0.63 – 1.55)	0.97	1.02 (0.65 – 1.59)	0.95
Divorced/widowed	252 (68.5%)	116 (31.5%)	1.44 (1.09 – 1.91)	0.01	1.55 (1.15 – 2.08)	0.004

*Patients missing WHO stage removed from analysis (n = 438).

^†^130 missing values.

^^^Adjusted for age, gender, marital status.

**Table 3:** **Socio-demographic, clinical characteristics and univariable/multivariable associations with late HIV disease presentation (CD4 count ≤ 350 cells/μl) at initial clinic visit**

**Characteristics**	Early presentation (CD4 > 350 cells/μl) N. (%)*	Late presentation (CD4 ≤ 350 cells/μl) N. (%)*	**Unadj OR (95% CI)**	***P***	**Adj OR** ^**^**^ **(95% CI)**	***P***
All Participants	297 (34.5%)	564 (65.5%)				
HIV Testing Source						
IPC	34 (47.2%)	38 (52.8%)	1			
VCT	263 (33.3%)	526 (66.7%)	1.79 (1.10 – 2.90)	0.002	2.15 (1.28 – 3.60)	0.005
Age						
50 years and under	264 (35.3%)	468 (64.7%)	1		1	
Over 50 years	33 (33.1%)	96 (66.9%)	1.64 (1.07 – 2.51)	0.002	1.72 (1.09 – 2.74)	0.02
Gender						
Female	190 (51.5%)	348 (48.5%)	1		1	
Male	107 (47.7%)	216 (52.3%)	1.10 (0.82 – 1.48)	0.51	1.01 (0.73 – 1.40)	0.94
Marital status^†^						
Married	150 (33.8%)	294 (66.2%)	1		1	
Never married	35 (41.2%)	50 (58.8%)	0.73 (0.45 – 1.17)	0.19	0.71 (0.44 – 1.16)	0.17
Divorced/widowed	79 (32.9%)	161 (67.1%)	1.04 (0.75 – 1.45)	0.82	0.96 (0.68 – 1.37)	0.83

*Patients missing CD4 count removed from analysis (n = 891).

^†^92 missing values.

^^^Adjusted for age, gender, marital status.

### Descriptive analysis

Of 1,752 patients included in analysis, 108 were from the IPC and 1,644 from VCT services. The median age was 38.6 years (range 38.0 – 39.1 years). The sample was nearly two-thirds female and more than half of the sample reported being married (Table 4). Nearly two-thirds of patients presented to the clinic with CD4 ≤ 350 cells/μl (mean CD4 304.0 cells/μl) and more than one quarter of patients presented in WHO clinical stage 3 or 4 (Table 4).

**Table 4:** **Socio-demographic and clinical characteristics of patients, by HIV testing source (IPC or VCT)**

**Characteristics**	**IPC N. (%)**	**VCT N. (%)**	**Total N. (%)**
All participants	108 (6.2%)	1,644 (93.8%)	1,752 (100%)
WHO clinical stage at initial clinic visit^*^			
1 or 2	75 (7.8%)	880 (92.2%)	955 (100.0%)
3 or 4	15 (4.2%)	344 (95.8%)	359 (100.0%)
CD4 count at initial clinic visit^**^			
>350 cells/mm^3^	45 (10.4%)	386 (89.6%)	431 (100.0%)
≤350 cells/mm^3^	27 (6.3%)	403 (93.7%)	430 (100.0%)
Mean CD4 count at initial clinic visit (cells/μl)	368.0	298.1	304.0
95% CI (cells/μl)	(305.0 – 431.0)	(280.1 – 316.2)	(286.6 – 321.4)
Age			
50 years and under	80 (5.4%)	1,405 (94.6%)	1,485 (100.0%)
Over 50 years	28 (10.5%)	239 (89.5%)	267 (100.0%)
Gender			
Female	73 (6.5%)	1,046 (93.5%)	1,119 (100.0%)
Male	35 (5.5%)	598 (94.5%)	633 (100.0%)
Marital status^***^			
Married	73 (8.1%)	830 (91.9%)	903 (100.0%)
Never married	4 (2.1%)	184 (97.9%)	188 (100.0%)
Divorced/widowed	23 (4.7%)	464 (95.3%)	487 (100.0%)

^*^438, ^**^891, ^***^174 missing values.

### Multivariable analysis

A multivariable analysis was conducted including all variables studied in the univariable analysis. Patients who tested HIV positive via VCT services had more than two times higher odds of presenting to initial clinic visit in WHO clinical stage 3 or 4 of HIV infection than those who tested HIV positive during the IPC (adjusted OR 2.39, 95% CI 1.24 – 4.60, p = 0.01) (Table 2). Patients who tested HIV positive via VCT services had more than two times higher odds of presenting to initial clinic visit with CD4 ≤ 350 cells/μl than those who tested HIV positive during the IPC (adjusted OR 2.15, 95% CI 1.28 – 3.61, p = 0.005) (Table 3). Mean CD4 count at initial clinic visit among patients testing HIV positive via VCT was significantly lower than those who tested HIV positive during the IPC (Table 5).

**Table 5:** **Two sample t test with equal variances comparing mean CD4 count between IPC and VCT testing sources**

**HIV testing source**	**Obs**	**Mean CD4 count (cells/μl) (95% CI)**
IPC	72	368.0 (304.0 – 432.0)
VCT	789	298.1 (280.0 – 316.2)
Total	861	304.0 (286.6 – 321.4)

t = 2.184, d.f = 859, p = 0.03.

Male gender and being divorced or widowed were independently associated with presenting late to initial clinic visit at WHO clinical stage 3 or 4 (Table 2). Age over 50 years was independently associated with presenting late to initial clinic visit with CD4 ≤ 350 cells/μl (Table 3).

## Discussion

Age, gender and current marital status are all associated with late HIV presentation (WHO clinical stage 3 or 4 or CD4 ≤ 350 cells/μl) at first clinic appointment, which supports the findings of previous studies. Men had 1.4 times higher odds of presenting to clinic late in the course of HIV infection compared to women, and gender differences have been observed in other studies with regard to presentation to HIV clinic [15,16], as well as general outpatient health service utilisation [17,18]. Age over 50 years was also significantly associated with late HIV presentation, as shown in other studies [19], which may be due to a lack of knowledge of the disease and services available, or a low self-perceived risk of HIV in older age groups [9]. Increasing age has also been shown to have a negative impact on retention in care and overall treatment outcomes [20]. Not being married was associated with late presentation, as reported in previous studies [15].

More than two-thirds of patients who tested HIV positive via VCT services presented to initial clinic with CD4 ≤ 350 cells/μl and one-quarter of patients who tested HIV positive via VCT services presented to initial clinic with WHO clinical stage 3 or 4 of HIV infection, which is comparable to other studies in sub-Saharan Africa [16]. A significantly lower proportion of patients who tested HIV positive during the community-based IPC presented to their initial clinic visit late in the course of infection. The IPC may have identified patients earlier in the clinical course of HIV infection in the community because these individuals may be asymptomatic and have less perceived risk than those who self-attended VCT services. Community-based campaigns of HIV testing may therefore be able to reach individuals who are unaware of their HIV status and still at an early stage of infection. [21] While the rate of people ever having been tested for HIV has increased in Kenya, nearly half of people living with HIV still do not know they are infected [12]. These individuals may not be symptomatic, may not have adequate knowledge or understanding of HIV signs and symptoms, and/or may have low self-perceived risk and understanding of HIV transmission [22,23].

It is important to acknowledge the limitations of this study. Other important potential confounders such as socioeconomic status, HIV status of the spouse and time required to travel to clinic were not included in the paper-based patient record or EMR system and therefore could not be included and adjusted for in the analysis. Comparative analysis of the entire dataset and inclusion of all patients testing HIV positive via VCT services was also not possible due to missing data in the dataset. Missing data more broadly reflects the fact that many medical records are rarely kept up to date and complete in sub-Saharan Africa, making their use problematic for rigorous analysis and follow-up of outcomes [24].

During the IPC in September 2008, a significantly higher median CD4 count was recorded at the point of testing compared to those testing HIV positive from the IPC at initial clinic visit during this study, suggesting a lack of timely linkage between testing and care [11]. Improved referral mechanisms, particularly from community-based settings, are therefore also required to ensure timely and accountable linkage between testing and treatment services to ensure the gains of earlier testing are realised [2,3,25]. Few referral tools have been developed however and effective linkage between HIV testing and initiation of ART remains a challenge [24,26]. Little systematic data exist on the proportion of people living with HIV who are linked to care [26,27] and, once linked to care, there are mixed findings regarding retention in care. In a systematic review by Rosen et al., no study was able to follow patients from testing to treatment if they were not already eligible for ART when diagnosed [24]. Reasons for poor linkage between HIV testing and treatment services are multi-factorial and include systemic factors such as inadequate training, lack of access to timely and reliable data (electronic or paper based) and individual patient factors such as financial costs and proximity or preference for certain health facilities [28-30]. Electronic medical record (EMR) systems could support linkage of patients from testing to treatment services and should be evaluated further in real-time program settings. EMR systems may also help to improve the quality of data collected, as well as support provision of clinical care and ensure individuals are monitored and retained throughout the HIV care and treatment continuum.

## Conclusions

The community-based IPC identified HIV positive individuals earlier in the course of HIV infection, compared to voluntary counselling and testing services. The IPC may have identified patients earlier in the clinical course of HIV infection in the community because these individuals may be asymptomatic and have less perceived risk than those who self-attended VCT services. Such community-based campaigns of HIV testing may be able to assist countries to achieve earlier testing and initiation of ART in the course of HIV infection. A significantly higher median CD4 count was recorded at the point of testing during the IPC compared to those testing HIV positive from the IPC at initial clinic visit. Improving referral mechanisms and strengthening linkages between HIV testing and treatment services remain a challenge and electronic medical record (EMR) systems may support monitoring of patients throughout the HIV care and treatment continuum.

## References

[1] UNAIDS. (2013). Global Report: UNAIDS report on the global AIDS epidemic 2013. Joint United Nations Programme on HIV/AIDS (UNAIDS).

[2] WHO. (2010). Towards Universal access: Scaling up priority HIV/AIDS interventions in the health sector. Progress Report 2010. World Health Organization WHO), Joint United Nations Programme on HIV/AIDS (UNAIDS), UN Childrens' Agency (UNICEF). Retrieved from http://www.who.int/

[3] UNAIDS. (2012). Treatment 2015. Joint United Nations Programme on HIV/AIDS (UNAIDS). 12349391

[4] Badri M, Lawn SD, Wood R (2006). Short-term risk of AIDS or death in people infected with HIV-1 before antiretroviral therapy in South Africa: a longitudinal study. Lancet.

[5] Castilla J, Sobrino P, de la Fuente L, Noguer I, Guerra L, Parras F (2002). Late diagnosis of HIV infection in the era of highly active antiretroviral therapy: consequences for AIDS incidence. Aids.

[6] Sabin CA, Smith CJ, Gumley H, Murphy G, Lampe FC, Phillips AN (2004). Late presenters in the era of highly active antiretroviral therapy: uptake of and responses to antiretroviral therapy. Aids.

[7] Leisegang R, Cleary S, Hislop M, Davidse A, Regensberg L, Little F (2009). Early and late direct costs in a Southern African antiretroviral treatment programme: a retrospective cohort analysis. Plos Med.

[8] Granich RM, Gilks CF, Dye C, De Cock KM, Williams BG (2009). Universal voluntary HIV testing with immediate antiretroviral therapy as a strategy for elimination of HIV transmission: a mathematical model. Lancet.

[9] Girardi E, Sabin CA, Monforte AD (2007). Late diagnosis of HIV infection: epidemiological features, consequences and strategies to encourage earlier testing. J Acquir Immune Defic Syndr.

[10] Bunnell R, Mermin J, De Cock KM (2006). HIV prevention for a threatened continent: implementing positive prevention in Africa. Jama.

[11] Lugada E, Millar D, Haskew J, Grabowsky M, Garg N, Vestergaard M (2010). Rapid implementation of an integrated large-scale HIV counseling and testing, malaria, and diarrhea prevention campaign in rural Kenya. PLoS One.

[12] NASCOP. (2013). Kenya AIDS Indicator Survey 2012. National AIDS and STI Control Program, Ministry of Health Kenya.

[13] Uamuzi Bora, About Uamuzi Bora [https://www.uamuzibora.org], (2014).

[14] Ministry of Medical Services, NASCOP. (2011). Guidelines for antiretroviral therapy in Kenya. 4th edition 2011. Ministry of Medical Services, National AIDS and STI Control Program

[15] Quigley MA, Morgan D, Malamba SS, Mayanja B, Okongo MJ, Carpenter LM (2000). Case–control study of risk factors for incident HIV infection in rural Uganda. J Acquir Immune Defic Syndr.

[16] Kigozi IM, Dobkin LM, Geng EH, Emenyonu NI, Bangsberg DR, Hahn JA (2009). Late-disease stage at presentation to an HIV clinic in the era of free antiretroviral therapy in Sub-Saharan Africa. J Acquir Immune Defic Syndr.

[17] Nabyonga J, Desmet M, Karamagi H, Kadama PY, Omaswa FG, Walker O (2005). Abolition of cost-sharing is pro-poor: evidence from Uganda. Health Policy Plan.

[18] Bertakis, K., Azari, R., & Helms, L. (2000). Gender differences in the utilization of health care services. Journal Family Practice. 10718692

[19] Bonjour MA, Montagne M, Zambrano M, Molina G, Lippuner C, Wadskier FG (2008). Determinants of late disease-stage presentation at diagnosis of HIV infection in Venezuela: a case-case comparison. AIDS Res Ther.

[20] Ekouevi DK, Balestre E, Ba-Gomis F-O, Eholie SP, Maiga M, Amani-Bosse C (2010). Low retention of HIV-infected patients on antiretroviral therapy in 11 clinical centres in West Africa. Tropical Med Int Health.

[21] Suthar AB, Ford N, Bachanas PJ, Wong VJ, Rajan JS, Saltzman AK (2013). Towards universal voluntary HIV testing and counselling: a systematic review and meta-analysis of community-based approaches. Plos Med..

[22] Ngatu NR, Hirota R, Eitoku M, Muzembo BA, Nishimori M, Kuramochi M, et al. Perception of the risk of sexual transmission of HIV among Congolese and Japanese university students, Environ Health Prev Med. 2011. doi:10.1007/s12199-011-0232-z. 10.1007/s12199-011-0232-z PMC3342628 21861117

[23] Puffer ES, Meade CS, Drabkin AS, Broverman SA, Ogwang-Odhiambo RA, Sikkema KJ (2011). Individual- and family-level psychosocial correlates of HIV risk behavior among youth in rural Kenya. AIDS Behav.

[24] Rosen S, Fox MP (2011). Retention in HIV care between testing and treatment in Sub-Saharan africa: a systematic review. Plos Med.

[25] UNAIDS. (2011, August 19). Getting To Zero: UNAIDS 2011-2015 Strategy. Unaids.org. Retrieved February 1, 2014, from http://www.unaids.int

[26] Wynberg E, Cooke G, Shroufi A, Reid SD, Ford N (2014). Impact of point-of-care CD4 testing on linkage to HIV care: a systematic review. J Int AIDS Soc.

[27] Kranzer K, Govindasamy D, Ford N, Johnston V, Lawn S D. Quantifying and addressing losses along the continuum of care for people living with HIV infection in sub-Saharan Africa: a systematic review, J Int AIDS Soc. 15, 2013. doi:10.7448/ias.15.2.17383. 10.7448/IAS.15.2.17383 PMC3503237 23199799

[28] Nsigaye R, Wringe A, Roura M, Kalluvya S, Urassa M, Busza J (2009). From HIV diagnosis to treatment: evaluation of a referral system to promote and monitor access to antiretroviral therapy in rural Tanzania. J Int AIDS Soc.

[29] Decroo T, Panunzi I, Das Dores C, Maldonado F, Biot M, Ford N (2009). Lessons learned during down referral of antiretroviral treatment in Tete, Mozambique. J Int AIDS Soc.

[30] Nordberg E, Holmberg S, Kiugu S (1996). Exploring the interface between first and second level of care: referrals in rural Africa. Trop Med Int Health.

